# Estimation of Amino Acids

**Published:** 1914-06

**Authors:** 


**Estimation of Amino Acids.** P. J. Cammidge, Lancet, Nov. 8, 1913, states that the Malfatti method gives the ammonia plus amino-acid N, the Folin method, the ammonia N alone. Hence, by substraction, the amino-acid N can be determined. In diabetes, the oxidation of amino-acids is interfered with.

